# B Vitamin and/or n-3 Fatty Acid Supplementation and Health-Related Quality of Life: Ancillary Findings from the SU.FOL.OM3 Randomized Trial

**DOI:** 10.1371/journal.pone.0084844

**Published:** 2014-01-17

**Authors:** Valentina A. Andreeva, Clotilde Latarche, Serge Hercberg, Serge Briançon, Pilar Galan, Emmanuelle Kesse-Guyot

**Affiliations:** 1 Nutritional Epidemiology Research Unit, Sorbonne-Paris-Cité, University of Paris XIII, French Institute of Health and Medical Research, National Institute of Agronomic Research, National Conservatory of Arts and Crafts, Bobigny, France; 2 University Hospital Center, Department of Clinical Epidemiology and Evaluation, Nancy, France; 3 Université de Lorraine, Université Paris Descartes, Nancy, France; 4 Department of Public Health, Avicenne Hospital, Bobigny, France; CUNY, United States of America

## Abstract

**Background:**

Despite growing attention to nutrition and quality of life in cardiovascular disease survivors, the impact of dietary factors according to disease type or to quality of life domain is poorly understood. We investigated the effects of B vitamin and/or n-3 fatty acid supplementation on health-related quality of life among survivors of stroke, myocardial infarction, or unstable angina.

**Methods:**

We performed ancillary analyses of the SU.FOL.OM3 trial (2003–2009; France). In total, 2,501 men (mean age = 61 y) and women (mean age = 63 y) were randomized in a 2×2 factorial design to: 1) 0.56 mg 5-methyl-tetrahydrofolate, 3 mg vitamin B6, 0.02 mg vitamin B12; 2) 600 mg eicosapentaenoic and docosahexaenoic acids in a 2∶1 ratio; 3) B vitamins and n-3 fatty acids combined; or 4) placebo. Health-related quality of life was evaluated at follow-up with the Medical Outcomes Study 36-Item Short Form Health Survey. Data from 2,029 individuals were used in this analysis.

**Results:**

After 3.1±0.4 y, no effects of supplementation with either B vitamins or n-3 fatty acids on quality of life (physical or mental health domains) were found. However, participants receiving B vitamins had slightly more activity limitations due to emotional problems compared with those not receiving B vitamins (mean difference = 3.8; 95% CI: 0.4, 7.1). A significant interaction of treatment by prior disease revealed an inverse association between n-3 fatty acids and vitality among myocardial infarction survivors (mean difference = 2.9; 95% CI: 0.5, 5.2).

**Conclusions:**

There were no beneficial effects of supplementation with relatively low doses of B vitamins or n-3 fatty acids on health-related quality of life in cardiovascular disease survivors. The adverse effects of B vitamins on activity limitations and of n-3 fatty acids on vitality among individuals with prior myocardial infarction merit confirmation.

## Introduction

The life span increase has amplified the prevalence of cardiovascular diseases (CVD) and age-related comorbidities [1], [2]. Thus, evaluation of health-related quality of life (QOL) constitutes an indispensable public health strategy especially with respect to the elderly [3] and it might also be a decisive factor when establishing healthcare priorities [4]. QOL encompasses one's physical and psychological state in a given sociocultural context [5], while health-related QOL reflects disease-induced changes in physical and psychosocial functioning [6]. In the United Kingdom, for example, one of the key guidelines advanced by the National Institute for Health and Care Excellence (NICE) of the Department of Health pertains to the need for a standardized QOL assessment in research with children and adults [7]. Health-related QOL and measures of quality-adjusted life years are also essential endpoints when evaluating intervention effects [8] and the cost-effectiveness of randomized controlled trials (RCT) [9]. In general, CVD survivors have reduced health-related QOL compared with the general population [10].

Optimal nutrition and physical activity are critical QOL determinants [11]. RCTs involving dietary interventions document beneficial effects on QOL among convalescing elderly [12] and heart failure patients [13], [14]. B vitamins, for example, are implicated in various neurophysiological functions, given their role in one-carbon metabolism, nucleic acid synthesis, methylation, and the fact that even mild deficiencies could lead to brain function impairment [15]. However, a 1-year RCT with daily supplementation with folic acid, vitamins B6 and B12 found no effect on health-related QOL among adults with mild cognitive impairment [16], while daily injection with 1 mg vitamin B12 over 4 weeks was positively associated with QOL among elderly with possible vitamin B12 deficiency [17].

The biological plausibility for long-chain n-3 polyunsaturated fatty acids (PUFA) to affect QOL [18] has also been advanced, given their role in neural membrane stability [19] and neuro-inflammation regulation [20]. Positive associations of fish consumption with mental [21] and physical health indices [22] have been reported. The positive association between serum phospholipid concentrations of eicosapentaenoic acid (EPA) and the physical domain was considered plausible given reduced synthesis of inflammatory series-2 prostaglandins and series-4 leukotrienes in favor of the less inflammatory series-3 prostaglandins and series-5 leukotrienes synthesized from EPA [23]. Prospective studies [24] or RCTs have been less compelling, possibly because of methodological heterogeneity. RCTs have reported beneficial effects of n-3 PUFA on mood profiles in healthy adults [25], on physical and mental indices in depressed elderly females [26], on global health, physical and social functioning among lung cancer patients [27], and on physical health in elderly with mild cognitive impairment [28]. However, a general elderly population RCT found no effects on overall QOL of a 13- or 26-week supplementation with two different doses of EPA and docosahexaenoic acid (DHA) [29].

Presently, QOL-focused dietary interventions with CVD survivors are scarce. One RCT with ischemic stroke patients observed no effects on QOL following a 12-week supplementation with 0.7 g DHA and 0.3 g EPA [30]. Considering the insufficient evidence, herein we assess the medium/long-term effects of B vitamin and/or n-3 PUFA on health-related QOL in individuals with CVD history, using data from the Supplementation With Folate, Vitamins B6 and B12 or Omega-3 Fatty Acids (SU.FOL.OM3) RCT. Given evidence of sex-specific differences in QOL outcomes (e.g., emotion, sleep, energy, pain and mobility) among stroke and myocardial infarction survivors [31]–[33], we hypothesized effect modification by sex. Also, each CVD type entails unique physical and psychological sequelae leading to a differential impact on QOL [34]. Hence, we also hypothesized effect modification by type of prevalent CVD.

## Methods

### Ethics statement

The SU.FOL.OM3 protocol was approved by the Consultation Committee for the Protection of Participants in Biomedical Research of the Paris-Cochin Hospital and by the French National Information and Citizen Freedom Committee. Written informed consent was provided by each participant [35].

### Study design and participants

**Figure 1:**
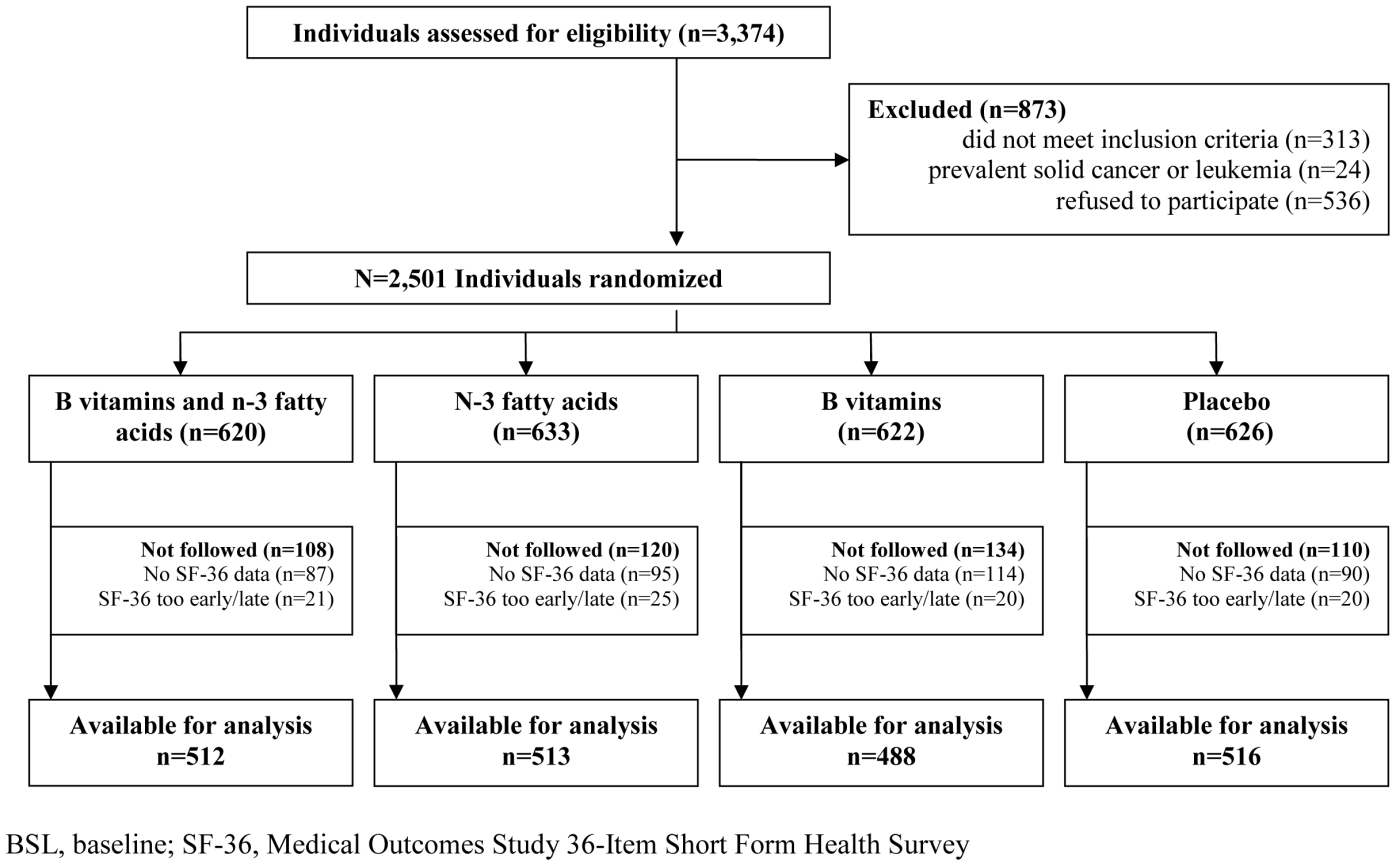
Flow chart of the study participants.

The SU.FOL.OM3 secondary prevention, double-blind RCT was conducted between February 1, 2003 and July 1, 2009 (Current Controlled Trials registration # ISRCTN41926726, http://www.controlled-trials.com/ISRCTN41926726) [35], [36]. Individuals aged 45–80 y who had experienced an acute myocardial infarction, unstable angina, or ischemic stroke within the preceding 12 months were eligible for recruitment via a network of 417 physicians throughout France (Figure 1). Individuals with non-CVD pathology (solid cancer, leukemia) and with an expected survival <5 y were ineligible [36]. Sample size calculation was informed by a comprehensive literature review and was based on an estimated CVD risk of 0.087 in the placebo group [35]. The median duration of supplementation was 4.7 y and the trial's primary outcomes were recurrent myocardial infarction, stroke, and CVD mortality. The protocol and principal findings are available elsewhere [35], [36].

### Randomization and intervention

Using computerized block randomization (block size = 8) with stratification by sex, age (45–54, 55–64, and 65–80 y), prior CVD, and recruitment center, the statistics team assigned 2,501 participants in a 2×2 factorial design to one of four groups: 1) 0.56 mg 5-methyl-tetrahydrofolate, 3 mg vitamin B6 (pyridoxine) and 0.02 mg vitamin B12 (cyanocobalamin); 2) 600 mg EPA and DHA in a 2∶1 ratio; 3) B vitamins and n-3 PUFA combined; or 4) placebo. The supplements were given as two capsules to be taken once daily and were provided free of charge by Merck Eprova AG (5-methyl-tetrahydrofolate; Schaffhausen, Switzerland), Roche Laboratories (vitamins B6 and B12; Basel, Switzerland), and Pierre Fabre Laboratories (n-3 PUFA; Ramonville, France). All capsules were made of gelatin manufactured by Catalent Pharma Solutions (Beinheim, France; Swindon, UK). The capsules were manufactured exclusively for the study and were not commercially marketed. Median time between the acute CVD event and randomization was 101 days [35]. The participants were given sufficient supplementation for one year and were re-examined and re-supplied at annual follow-up visits.

### Outcome assessment

Health-related QOL was evaluated with the validated French version of the Medical Outcomes Study 36-Item Short Form Health Survey (SF-36). The QOL protocol was added after the start of data collection. The SF-36 captures the following eight domains: physical functioning, role limitations due to physical problems, bodily pain, general health perceptions, vitality, social functioning, role limitations due to emotional problems, and general mental health [37], [38]. All except one item (self-perceived health) were used in scoring these eight measures (score range: 0 = worst, 100 = best). Next, summary measures of physical health (PCS, physical component summary, including physical functioning, role limitations due to physical problems, bodily pain, and general health perceptions) and mental health (MCS, mental component summary, including vitality, social functioning, emotional problems, and general mental health) were computed using established, normed reference values (expected mean = 50.0, SD = 10.0) [39]. The SF-36 was administered at Years 3 and 5 of follow-up.

In total, 2,100 participants completed the SF-36 at Year 3; 1,329 - at Year 5; and 1,263 - at both waves. Consistent with our objective to assess the medium/long-term supplementation effect, we retained participants with QOL data provided between 2.5 (ie, Year 3) and 5 y post-baseline. Individuals with incomplete QOL data were excluded. Thus, we retained 1,978 individuals who had completed the SF-36 at Year 3 and 51 individuals who had completed it at Year 5 (with missing or incomplete Year 3 data).

### Covariates

Information about age, sex, marital status (married/cohabiting versus other), educational level (<high school, high school diploma, or post-secondary education), employment status (employed versus other), birthplace (birth in France or abroad), tobacco use (current, former, or never smoker), heavy alcohol use (yes/no; defined as >20 g/d for women and >30 g/d for men), body mass index (BMI, kg/m^2^), blood pressure, and type of prevalent CVD was collected at baseline. Blood pressure was measured by trained staff using a semi-automatic device (Digital blood pressure monitor Omron UA-787; Omron Corp., Kyoto, Japan), following a standardized protocol [40]. Baseline (n = 2,501) and follow-up (n = 2,147 at 1-year follow-up, and n = 1,160 at the end of the trial) concentrations of serum folate, serum vitamin B12, plasma vitamin B6, and plasma EPA+DHA (% of fatty acids) were also assessed. Blood samples were obtained after a minimum of a 5-hour fast and were treated/stored and all biomarkers measured according to strict protocol guidelines [35]. Fatty acid composition of plasma lipids was determined by gas chromatography in a random sample of 682 individuals at baseline and at 1-year follow-up. During follow-up, we evaluated treatment compliance (taking ≥80% of the assigned supplements) and possible adverse effects [35].

### Statistical analysis

The factorial design necessitated the assessment of interaction between the treatments. These tests did not reveal any effect modification regarding PCS (p>0.92) or MCS (p>0.35). Hence, we evaluated the effect of B vitamins (alone or combined with n-3 PUFA) and the effect of n-3 PUFA (alone or combined with B vitamins) on health-related QOL. Baseline characteristics and group comparability were explored with chi-squared tests, unpaired *t* tests, and Wilcoxon Rank Sum tests. We evaluated the bivariate associations of QOL with each covariate and all significant variables were retained for multivariate modeling via analysis of covariance (ANCOVA). We aimed to fit well-defined models, adjusted for the most pertinent covariates in order to minimize the potential for type I error. We also performed tests for interaction between sex and prior CVD, respectively, and supplement type. For the bivariate analysis and for the tests for interaction, the significance level was set at 0.20, whereas for the principal ANCOVA models the significance level was 0.05 (2-sided). Analyses were conducted with SAS software (version 9.2; SAS Institute, Inc.) without interfering with the *intent-to-treat* principle regarding supplement allocation.

## Results

The final sample consisted of 2,029 individuals (81.1% of the SU.FOL.OM3 cohort) with QOL data from a single timepoint after a mean follow-up of 3.1±0.4 y. Baseline characteristics by treatment type are summarized in Table 1. The mean age was 61.2±8.8 y and men constituted 79.6% of the sample. Treatment groups were well balanced regarding all baseline characteristics except for a slightly higher proportion of heavy alcohol users among those not receiving B vitamins (p<0.05). Compared with individuals excluded from the analysis (n = 472), those included had higher baseline concentrations of folate, vitamin B6 and EPA+DHA, and were less likely to be foreign-born and current smokers.

**Table 1:** Baseline characteristics by supplement allocation (N = 2,029)^a^.

	B vitamins					N-3 fatty acids				
	Yes (n = 1,000)		No (n = 1,029)		*P* b	Yes (n = 1,025)		No (n = 1,004)		*P* b
*Demographic*										
Age, mean (SD), y	61.1	(8.8)	61.4	(8.8)	*0.42*	61.1	(9.1)	61.4	(8.6)	*0.46*
Female, No. (%)	199	(19.9)	215	(20.9)	*0.58*	206	(20.1)	208	(20.7)	*0.73*
Married/cohabiting, No. (%)	722	(72.2)	753	(73.2)	*0.71*	737	(71.9)	738	(73.5)	*0.67*
Post-secondary education, No. (%)	148	(14.8)	170	(16.5)	*0.29*	162	(15.8)	156	(15.5)	*0.80*
Employed, No. (%)	361	(36.1)	367	(35.7)	*0.75*	382	(37.3)	346	(34.5)	*0.14*
Foreign-born, No. (%)	111	(11.1)	107	(10.4)	*0.61*	112	(10.9)	106	(10.6)	*0.79*
*Behavioral [No. (%)]*										
Current smoker	97	(9.7)	100	(9.7)	*0.99*	99	(9.7)	98	(9.8)	*0.99*
Former smoker	615	(61.5)	623	(60.5)	*0.65*	615	(60.0)	623	(62.1)	*0.50*
Heavy alcohol use^c^	241	(24.1)	287	(27.9)	***0.03***	264	(25.8)	264	(26.3)	*0.87*
*Clinical (mean, SD)*										
Body mass index (kg/m^2^)	27.6	(4.1)	27.4	(3.8)	*0.42*	27.5	(4.1)	27.5	(3.8)	*0.95*
Diastolic blood pressure (mmHg)	83.2	(12.2)	83.0	(12.0)	*0.66*	83.3	(11.8)	82.9	(12.3)	*0.45*
Systolic blood pressure (mmHg)	132.7	(21.0)	133.3	(21.0)	*0.52*	133.0	(21.1)	133.0	(20.9)	*0.93*
*Biological (median, interquartile range)*										
Serum folate (ng/ml)	6.8	(3.4)	6.9	(3.6)	*0.98*	6.8	(3.5)	6.9	(3.5)	*0.43*
Plasma vitamin B_6_ (nmol/L)	37.8	(23.6)	38.8	(26.0)	*0.53*	38.7	(24.3)	38.1	(24.5)	*0.71*
Serum vitamin B_12_ (pg/ml)	363.0	(161.0)	372.0	(172.0)	*0.20*	363.0	(170.0)	373.0	(165.0)	*0.18*
Plasma EPA+DHA^d^ (%)	3.8	(2.0)	4.0	(2.1)	*0.26*	3.8	(2.1)	3.9	(2.0)	*0.76*
*Cardiovascular disease history [No. (%)]*										
Myocardial infarction	469	(46.9)	470	(45.7)	*0.58*	476	(46.4)	463	(46.1)	*0.88*
Unstable angina	282	(28.2)	302	(29.4)	*0.57*	301	(29.4)	283	(28.2)	*0.56*
Ischemic stroke	249	(24.9)	257	(25.0)	*0.97*	248	(24.2)	258	(25.7)	*0.43*

SI conversion: folate - to convert to nmol/L, multiply by 2.266; vitamin B_6_ - to convert to ng/mL, divide by 4.046; vitamin B_12_ - to convert to pmol//L multiply by 0.7378.

^a^ Sample with available SF-36 quality of life data provided 2.5–5.0 y post-baseline.

^b^ Based on chi-square tests, Student t-tests, or Wilcoxon Rank Sum tests, as appropriate.

^c^ Defined as >20 g/d for women and >30 g/d for men.

^d^ EPA, eicosapentaenoic acid; DHA, docosahexaenoic acid.

QOL score distribution and sample characteristics by prior CVD are presented in Table 2. Most baseline characteristics as well as PCS, MCS, and all eight QOL variables were significantly different across CVD type. Higher QOL was noted in myocardial infarction survivors, while lower QOL was observed among stroke survivors. The mean PCS score was 46.5 (SD = 9.2), the mean MCS score was 47.5 (SD = 9.9), and their distribution was near normal.

**Table 2:** Sample characteristics and SF-36 quality of life measures by type of prior cardiovascular disease (N = 2,029).

	Myocardial infarction (n = 939)		Unstable angina (n = 584)		Ischemic stroke (n = 506)		*P* a
*Treatment allocation*							
B vitamins, No. (%)	469	(49.9)	282	(48.3)	249	(49.2)	*0.82*
n-3 fatty acids, No. (%)	476	(50.7)	301	(51.5)	248	(49.0)	*0.70*
*Baseline characteristics*							
Female, No. (%)	148	(15.8)	113	(19.4)	153	(30.2)	***<0.0001***
Age^b^, y	59.6	(8.5)	62.1	(8.9)	63.2	(9.0)	***<0.0001***
Married/cohabiting, No. (%)	701	(74.7)	412	(70.5)	362	(71.5)	***0.15***
Post-secondary education, No. (%)	153	(16.3)	99	(17.0)	66	(13.0)	***0.15***
Employed, No. (%)	408	(43.5)	186	(31.8)	134	(26.5)	***<0.0001***
Foreign-born, No. (%)	92	(9.8)	70	(12.0)	56	(11.1)	*0.39*
Current smoker, No. (%)	85	(9.1)	46	(7.9)	66	(13.0)	***0.01***
Former smoker, No. (%)	613	(65.3)	370	(63.4)	255	(50.4)	***<0.0001***
Heavy alcohol use^c^, No. (%)	263	(28.0)	142	(24.3)	123	(24.3)	***0.13***
Body mass index^b^ (kg/m^2^)	27.4	(3.8)	27.6	(4.0)	27.6	(4.2)	*0.57*
Diastolic blood pressure^b^ (mmHg)	81.0	(11.7)	83.1	(11.8)	87.1	(11.9)	***<0.0001***
Systolic blood pressure^b^ (mmHg)	128.5	(20.0)	134.8	(21.2)	139.4	(20.9)	***<0.0001***
Serum folate^d^ (ng/ml)	6.7	(3.5)	7.1	(3.5)	6.9	(3.4)	***0.16***
Plasma vitamin B_6_ d (nmol/L)	38.9	(24.6)	38.7	(25.1)	36.9	(23.9)	*0.35*
Serum vitamin B_12_ d (pg/ml)	363.0	(165.0)	368.5	(163.0)	375.5	(183.5)	*0.23*
Plasma EPA+DHA^d^^,^ e (%)	3.8	(2.1)	4.0	(2.1)	3.7	(1.9)	***0.04***
*SF-36 quality of life measures at follow-up* b							
Self-perceived health	50.5	(15.6)	50.3	(15.6)	50.3	(16.5)	*0.95*
Physical component summary (PCS)	47.8	(8.7)	46.6	(9.2)	44.0	(9.7)	***<0.0001***
Physical functioning	79.1	(21.1)	77.7	(21.7)	67.3	(27.4)	***<0.0001***
Role limitations due to physical problems	77.5	(34.5)	74.4	(36.3)	67.4	(38.6)	***<0.0001***
Bodily pain	71.3	(25.3)	68.7	(25.3)	64.7	(25.2)	***<0.0001***
General health	62.1	(18.6)	59.7	(19.2)	58.5	(19.5)	***0.001***
Mental component summary (MCS)	47.7	(9.8)	47.9	(9.3)	46.6	(10.5)	***0.07***
Vitality	55.6	(17.8)	55.8	(16.8)	51.7	(18.4)	***<0.0001***
Social functioning	80.5	(21.2)	79.6	(21.6)	74.9	(24.2)	***<0.0001***
Role limitations due to emotional problems	78.4	(35.9)	77.3	(36.0)	69.8	(39.2)	***<0.0001***
Mental health	68.0	(17.9)	68.2	(17.5)	65.5	(19.0)	***0.02***
Duration of supplementation (in years) at time of SF-36 administration^b^	3.1	(0.4)	3.1	(0.4)	3.1	(0.4)	*0.22*

^a^ Based on one-way ANOVA or Kruskal-Wallis tests, as appropriate.

^b^ Values are means (SD).

^c^ Defined as >20 g/d for women and >30 g/d for men.

^d^ Values are medians (interquartile range).

^e^ EPA, eicosapentaenoic acid; DHA, docosahexaenoic acid.

### Participant characteristics according to heath-related QOL scores

Bivariate analyses revealed significant positive associations between PCS and being male, employed, having higher education, a history of myocardial infarction, being married, a former smoker, a heavy drinker, having higher concentrations of folate, vitamin B6, vitamin B12, and EPA+DHA. Significant inverse associations were observed between PCS and being foreign-born, a current smoker, having a higher BMI, a history of ischemic stroke, higher systolic and diastolic blood pressure. Regarding MCS, the bivariate analysis revealed significant positive associations with being male, married, a former smoker, a heavy drinker, having higher systolic blood pressure, and higher folate concentrations. Significant inverse associations were observed between MCS and being foreign-born, a current smoker, and having a history of ischemic stroke.

The interaction between treatment type and history of ischemic stroke or myocardial infarction, respectively, was not significant regarding PCS or MCS (all p>0.30). There was significant interaction between allocation to B vitamins (yes/no) and history of unstable angina regarding MCS.

### B vitamin supplementation and health-related QOL

Mean QOL score distribution by supplement allocation is presented in Table 3. Individuals allocated to B vitamins exhibited lower mean scores regarding role limitations due to emotional problems (*t* test p = 0.11). None of the other bivariate associations reached statistical significance. Table 4 presents the main ANCOVA results (mean difference+95% CI) regarding the effect of B vitamin supplementation on health-related QOL – in the full sample and by type of prior CVD. As the interaction term with sex and B vitamin allocation was not significant (MCS: p>0.60; PCS: p>0.99), we did not conduct sex-specific analyses. Models were adjusted for sex, age, heavy alcohol use, CVD, and the interval between baseline and SF-36 administration. These analyses revealed more role limitations due to emotional problems in individuals allocated to B vitamins (mean difference = 3.8; 95% CI: 0.4, 7.1; p = 0.029). No other statistically significant findings emerged.

**Table 3:** SF-36 quality of life measures^a^ at follow-up by supplement allocation (N = 2,029).

	B vitamins					N-3 fatty acids				
	Yes (n = 1,000)		No (n = 1,029)		*P* b	Yes (n = 1,025)		No (n = 1,004)		*P* b
Self-perceived health	50.0	(15.7)	50.8	(15.9)	*0.27*	50.3	(16.4)	50.5	(15.2)	*0.72*
Physical component summary (PCS)	46.7	(9.1)	46.2	(9.4)	*0.28*	46.4	(9.4)	46.5	(9.0)	*0.77*
Physical functioning	76.1	(23.6)	75.4	(23.4)	*0.49*	75.8	(23.2)	75.8	(23.8)	*0.96*
Role limitations due to physical problems	74.0	(36.0)	74.2	(36.6)	*0.89*	73.7	(36.5)	74.5	(36.1)	*0.63*
Bodily pain	69.4	(25.6)	68.5	(25.2)	*0.45*	68.9	(26.0)	69.0	(24.8)	*0.97*
General health	61.0	(19.2)	60.1	(18.9)	*0.30*	60.5	(18.9)	60.5	(19.2)	*0.98*
Mental component summary (MCS)	47.3	(10.0)	47.7	(9.7)	*0.38*	47.4	(9.9)	47.6	(9.8)	*0.72*
Vitality	54.6	(18.2)	54.8	(17.3)	*0.83*	54.2	(18.0)	55.2	(17.5)	*0.18*
Social functioning	78.9	(22.7)	78.9	(21.8)	*0.99*	78.4	(22.4)	79.3	(22.1)	*0.34*
Role limitations due to emotional problems	74.6	(37.5)	77.2	(36.4)	*0.11*	76.0	(36.8)	75.9	(37.0)	*0.95*
Mental health	67.6	(18.5)	67.3	(17.6)	*0.71*	67.5	(18.5)	67.4	(17.7)	*0.91*

^a^ Values are means (SD).

^b^ Based on Student t-tests.

**Table 4:** Associations between medium/long-term supplementation with B vitamins or n-3 fatty acids and health-related quality of life in the full sample and by prior cardiovascular disease.

	Full sample (n = 2,029)		Myocardial infarction (n = 939)		Unstable angina (n = 584)		Ischemic stroke (n = 506)	
	B vitamins	n-3 fatty acids	B vitamins	n-3 fatty acids	B vitamins	n-3 fatty acids	B vitamins	n-3 fatty acids
Self-perceived health	0.9 (−0.5, 2.4)	0.3 (−1.2, 1.8)	0.6 (−1.5, 2.7)	1.1 (−1.0, 3.2)	1.6 (−1.0, 4.2)	0.7 (−1.9, 3.3)	0.8 (−2.4, 3.9)	−1.7 (−4.8, 1.5)
Physical component summary (PCS)	0.0 (−0.8, 0.8)	−0.0 (−0.9, 0.8)	0.0 (−1.1, 1.2)	0.0 (−1.1, 1.2)	0.1 (−1.4, 1.6)	−0.3 (−1.8, 1.2)	−0.1 (−1.9, 1.7)	0.3 (−1.5, 2.0)
Physical functioning	0.5 (−1.6, 2.5)	0.1 (−1.9, 2.1)	−0.2 (−3.0, 2.6)	0.7 (−2.1, 3.5)	2.1 (−1.3, 5.6)	−1.4 (−4.8, 2.1)	−0.1 (−5.0, 4.9)	0.7 (−4.2, 5.7)
Role limitations due to physical problems	1.0 (−2.4, 4.3)	0.6 (−2.7, 4.0)	1.5 (−3.2, 6.3)	1.3 (−3.4, 6.0)	0.3 (−5.8, 6.5)	0.8 (−5.4, 6.9)	1.4 (−5.8, 8.6)	−0.6 (−7.8, 6.6)
Bodily pain	0.0 (−2.3, 2.3)	−0.4 (−2.7, 1.9)	0.5 (−2.9, 3.9)	−0.2 (−3.6, 3.2)	−1.1 (−5.5, 3.3)	−1.6 (−5.9, 2.7)	0.4 (−4.3, 5.1)	1.0 (−3.6, 5.7)
General health	−0.1 (−1.9, 1.6)	−0.4 (−2.2, 1.3)	0.6 (−2.0, 3.1)	0.0 (−2.5, 2.6)	−1.3 (−4.6, 2.0)	0.1 (−3.2, 3.4)	−0.0 (−3.6, 3.6)	−1.6 (−5.3, 2.0)
Mental component summary (MCS)	0.4 (−0.5, 1.3)	0.3 (−0.6, 1.2)	0.6 (−0.7, 1.9)	0.9 (−0.4, 2.2)	−0.4 (−2.0, 1.2)	−0.1 (−1.7, 1.5)	1.1 (−0.8, 3.1)	−0.6 (−2.5, 1.4)
Vitality	0.6 (−1.0, 2.2)	0.9 (−0.7, 2.5)	0.8 (−1.6, 3.2)	2.9 (0.5, 5.2)**	−0.6 (−3.5, 2.3)	−0.8 (−3.7, 2.1)	1.5 (−1.9, 5.0)	−0.5 (−3.9, 3.0)
Social functioning	0.4 (−1.6, 2.5)	0.9 (−1.1, 2.9)	0.3 (−2.6, 3.2)	0.8 (−2.1, 3.6)	−1.4 (−5.1, 2.3)	0.7 (−3.0, 4.3)	2.9 (−1.5, 7.3)	1.1 (−3.3, 5.5)
Role limitations due to emotional problems	3.8 (0.4, 7.1)*	0.2 (−3.2, 3.5)	3.8 (−1.1, 8.7)	2.4 (−2.5, 7.2)	3.8 (−2.2, 9.8)	−1.6 (−7.6, 4.3)	4.3 (−2.9, 11.6)	−1.7 (−9.0, 5.5)
Mental health	−0.4 (−2.0, 1.3)	0.1 (−1.5, 1.7)	0.0 (−2.4, 2.5)	1.0 (−1.4, 3.5)	−1.7 (−4.7, 1.2)	−0.2 (−3.2, 2.7)	0.4 (−3.0, 3.8)	−1.2 (−4.6, 2.1)

Values are estimated mean difference (95% confidence interval) from ANCOVA models adjusted for sex, baseline age, heavy alcohol use, prior cardiovascular disease (full sample analyses), and interval between baseline and SF-36 administration.

p = 0.029;

p = 0.018.

Given the results of the preliminary bivariate models, we carried out sensitivity analyses with PCS and MCS as the respective dependent variables, adjusting for additional covariates. The PCS models were adjusted for marital status, education, birthplace, smoking status, BMI, systolic blood pressure, concentrations of folate, vitamins B6 and B12, and EPA+DHA. The MCS models were additionally adjusted for marital status, birthplace, smoking status, systolic blood pressure, and folate concentration. Again, no significant associations emerged (results not tabulated).

### n-3 PUFA supplementation and health-related QOL

Mean QOL score distribution by supplement group (Table 3) revealed a significant inverse association between allocation to receive n-3 PUFA and mean vitality scores (*t* test p = 0.18). ANCOVA results regarding the effect of n-3 PUFA supplementation are summarized in Table 4. These models were also adjusted for sex, age, heavy alcohol use, CVD, and the interval between baseline and SF-36 administration. The interaction term with sex and n-3 PUFA allocation was not significant regarding PCS (p>0.20) or MCS (p>0.89). In the full sample, no significant associations were observed. In the subsample with a history of myocardial infarction, lower vitality was found among those receiving n-3 PUFA (mean difference = 2.9; 95% CI: 0.5, 5.2; p = 0.018). Again, we carried out sensitivity analyses with PCS and MCS, adjusting for the same additional variables. No significant associations between n-3 PUFA allocation and either of the summary measures emerged (results not tabulated).

## Discussion

The present findings, derived from ancillary data analyses of the SU.FOL.OM3 trial, did not provide support for beneficial effects of a relatively long-term, low-dose supplementation with B vitamins or n-3 PUFA on health-related QOL among CVD survivors. However, those receiving B vitamins showed slightly more daily activity limitations due to emotional problems compared to those not receiving B vitamins. With respect to estimating the practical or clinical significance of our results, the mean difference between the groups (3.8 points) is slightly above the 3-point minimal important difference reported across different clinical populations [41], [42]. Nonetheless, it should be pointed out that there is no single correct way of estimating the minimal important difference [41], and a variety of anchor-based and distribution-based methods have been advanced [43].

It should be noted that compared to excluded participants, those included in the analyses had higher baseline concentrations of folate, vitamin B6 and EPA+DHA, which might have resulted in underestimation of the associations. Additionally, individuals with a history of myocardial infarction allocated to n-3 PUFA evinced lower vitality compared to those not receiving n-3 PUFA or those with different CVD history (mean difference = 2.9 points). This finding is unexpected given these nutrients' role in neuro-inflammation regulation [20]. Vitality scores were normally distributed and were higher among survivors of myocardial infarction or unstable angina than among stroke survivors. Authors caution that interpretation of health-related QOL outcomes in clinical trials is challenging because thresholds regarding each QOL domain might be disease-, severity- and nationality-specific [44]. Meanwhile, even small individual-level differences in QOL scores might have substantial importance at the population level [43].

A small RCT involving a 9-month, very high-dose supplementation with multiple micronutrients (including several B vitamins) has previously reported beneficial QOL effects (assessed by the EuroQoL heart failure instrument) in elderly patients with chronic heart failure [45]. However, RCTs with clinical (but not CVD) samples have documented either no QOL effects [16] or isolated beneficial effects on general health [17] following B vitamin treatment. Consistent with our findings, an RCT with ischemic stroke patients observed no effects on the SF-36 indices of a 12-week supplementation with 3 g/d fish oil containing 0.7 g DHA and 0.3 g EPA [30]. In that study, baseline and follow-up PCS and MCS scores were similar to those found among stroke survivors in our sample. A primary prevention RCT also did not find significant effects of a 26-week n-3 PUFA supplementation (1,800 mg/d EPA+DHA, 400 mg/d EPA+DHA, or placebo) among community-dwelling elderly [29].

A literature review revealed that age, symptom severity and the timing of assessment were major QOL determinants among myocardial infarction patients [46], whereas other reports indicate a lack of a linear association between time since the CVD event and health-related QOL [10]. An ancillary study of the GISSI-3 trial with acute myocardial infarction patients documented that at six weeks, the illness had an impact on most health-related QOL domains, whereas at six months there was a major impact on functional status. In addition, lower QOL scores were found among women, the elderly, and among patients with severe left ventricular dysfunction [47]. Other research with female myocardial infarction survivors showed a non-significant link between time since the event (range: 3 months −5 y) and QOL (especially physical health) indicators [48]. Another study with CVD survivors demonstrated that the detrimental effect on health-related QOL persisted over the 1-year follow-up, with no recovery observed in patients with acute myocardial infarction and a continued decline observed in congestive heart failure patients [34]. Research has also documented continued decline 3 months post-diagnosis with acute coronary syndrome regarding physical functioning, vitality, general health perceptions, and PCS [49]. There is evidence that different QOL domains improve at markedly varied rates post-CVD [50].

Among the multiple QOL measures, the one most commonly used in samples of CVD patients [51], [52] and demonstrating strong psychometric properties [53], [54] is the SF-36 [37]. Yet, factors related to nutrition have not been routinely incuded in QOL assessment [16], [55]. Additionally, the SF-36 is a generic measure and does not include disease-specific indices [56]. Whereas the SF-36 was administered twice (at Years 3 and 5 of follow-up, but not at baseline), only half of the cohort had completed it at both waves, preventing assessment of pre- and post-treatment differences, due to possible selection bias. Also, the lack of baseline QOL assessment likely has reduced impact given the success of the randomization. Another limitation of the study was the performance of ancillary analyses of a secondary outcome (of a dynamic nature) [52]. Our RCT was designed as a secondary CVD prevention trial and its main results showed that allocation to B vitamins or to n-3 PUFA had no effect on major vascular events [35]. A further limitation was the reliance on ANCOVA even though several of the QOL measures were not normally distributed. Log-transformations to smooth out these distributions were not feasible given the possible value ranges. However, departures from the normality assumption generally have reduced impact in large samples, such as ours. Moreover, authors advocate the use of the same method of analysis for all SF-36 domains [57]. Another limitation concerns the performance of multiple comparisons while retaining the significance levels set *a priori* in the trial protocol, thus preserving statistical power. Nonetheless, we cannot rule out potential adverse effects of B vitamins on activity limitations due to emotional problems or of n-3 PUFA on vitality among survivors of myocardial infarction. However, it is possible that these inverse associations as well as the lack of other significant findings might be due to the low treatment doses used, to other study design aspects, or to chance. Next, only limited data on dietary supplementation outside the trial were available, preventing an accurate account for that covariate. The randomization procedure, however, was regarded as successful in balancing the treatment groups across measured/unmeasured residual constructs.

A distinctive feature of the trial was the relatively low treatment doses, reflecting interest in the impact of nutritional and not pharmacologic supplementation. The absence of folic acid fortification in France has implications for the generalizability of our findings. Strengths of the study include the relatively large sample and the use of 5-methyl-tetrahydrofolate, which is the most abundant natural folate form. Unlike folic acid, 5-methyl-tetrahydrofolate is not likely to mask vitamin B12 deficiency via unmetabolized folic acid circulation. Treatment compliance (defined as taking ≥80% of the assigned supplements) was assessed by self-reports via the follow-up questionnaires and by blood concentrations of B vitamins and n-3 PUFA. As previously documented [35], approximately 86% of the participants who returned a completed follow-up questionnaire self-reported being compliant with the allocated treatment, with compliance being similar across the four treatment groups. At the end of the trial, treatment with B vitamins was associated with a 146% increase in serum folate, a 116% increase in plasma vitamin B6, and a 35% increase in serum vitamin B12. In turn, treatment with EPA+DHA was associated with a 37% increase in median plasma concentrations of n-3 PUFA at 1-year follow-up compared with the placebo group [35]. Whereas a number of validated analytical methods for the assessment of n-3 PUFA status exist (each presenting strengths and weaknesses), a recent literature review highlighted that, at present, there is no agreed upon standard procedure for fatty acid analysis [58]. Plasma n-3 PUFA, for example, reflect short-term dietary fatty acid intake, whereas erythrocyte n-3 PUFA undergo a slow turnover rate, thus reflecting long-term fatty acid intake [59].

In conclusion, our results do not support dietary B vitamin or n-3 PUFA supplementation for health-related QOL promotion among CVD survivors. Replication of the models in larger cohorts with multiple QOL assessments is necessary, as is inclusion of the present results in future meta-analyses. The preliminary evidence of adverse effects of B vitamins on activity limitations due to emotional problems, and of n-3 PUFA on vitality among individuals with prior myocardial infarction merits confirmation before drawing broad conclusions.
